# The Importance of the Neutrophil–Lymphocyte Ratio (NLR) and the Platelet–Lymphocyte Ratio (PLR) as Biomarkers for Differentiating Complicated and Uncomplicated Appendicitis

**DOI:** 10.3390/diagnostics14242777

**Published:** 2024-12-11

**Authors:** Michail Anastasakis, Ioannis Trevlias, Konstantinos Farmakis, Ioannis Valioulis

**Affiliations:** 1st Department of Pediatric Surgery, Aristotle University of Thessaloniki, 54124 Thessaloniki, Greecefarmakiskonstantinos@gmail.com (K.F.)

**Keywords:** appendicitis, complicated, uncomplicated, pediatric population, Neutrophil-to-Lymphocyte ratio, Platelet-to-Lymphocyte ratio

## Abstract

Background: This specific study evaluates the accuracy of two ratios, Neutrophil-to-Lymphocyte (N/L) and Platelet-to-Lymphocyte (P/L), as inflammatory markers on differentiating simple and complicated appendicitis preoperatively. Methods: The medical records of 341 children, up to 16 years old, with suspected acute appendicitis (AA) who underwent appendectomy, laparoscopic or open, between January 2020 and December 2022, in our department, were retrospectively reviewed. Routine blood exams and the demographic details were obtained. The area under the curve (AUC), sensitivity, and specificity of the above-mentioned markers regarding the differentiation of simple and complicated appendicitis, based on the statistical program SPSS, were calculated. Results: From the total of 341 patients, a percentage of 27.5% were related to perforated appendicitis, such as necrosis or the rupture of the appendiceal wall. A significant association was observed between perforated appendicitis and NLR values > 7.92, with concomitant sensitivity 62.5% and specificity 74.2%. Retrospectively, for PLR values > 180.57, the sensitivity was 61.1% and specificity 68.9%. For these models, the areas under the curve were 0.736 and 0.696. Conclusions: Our study revealed a significant association of N/L and P/L ratios with acute perforated appendicitis. Future studies should validate the use of these markers on this disease.

## 1. Introduction

The earliest medical accounts of a condition similar to acute appendicitis date back approximately 500 years, referred to as “perityphlitis” [1]. In 1886, Fitz clarified that this perityphlitis arises from appendix inflammation and introduced the term “appendicitis” [2]. It is a blind intestinal diverticulum that contains masses of lymphatic tissue and measures about 4.5 cm in children, but its length can range from 0.3 to 33 cm [3]. For many years, most pediatric and adult surgeons have considered it important to classify the disease according to the phase of inflammation observed during surgery. This classification helps determine the appropriate use of antibiotics and how patients are managed after surgery. Although various organizations have tried to establish a classification system, none have been widely accepted. Various scoring systems have been documented to aid in diagnosing suspected acute appendicitis, independent of clinical examination. The most commonly used are the Alvarado Score and the Appendicitis Inflammatory Response (AIR) Score [4,5]. Meeting more than five criteria of the Alvarado Score provides a sensitivity of 99%, but only 43% specificity; this improves to 81% and 82% with over seven criteria. This score is particularly useful for ruling out the diagnosis. In contrast, the AIR score shows high sensitivity and specificity (99%) when more than eight criteria are met [6]. Thus, scoring tools are not routinely used.

According to the international literature, acute appendicitis is classified into two primary categories—uncomplicated and complicated appendicitis—based on clinical criteria or histopathological findings. The World Society of Emergency Surgery (WSES) defines complicated appendicitis as encompassing the gangrenous phase, which involves necrosis, as well as the rupture of the organ wall. Conversely, uncomplicated appendicitis is characterized by the acute inflammation and hyperemia of the wall, along with the formation of an abscess prior to the onset of necrosis. This classification serves as the fundamental framework for categorizing cases in the present statistical study [7].

For decades, it has been established that no single marker provides high sensitivity and specificity for diagnosing acute appendicitis. The white blood cell count (WBC) and C-reactive protein (CRP) are considered the primary indicators, commonly used in hospitals. They are essential for phagocytosis and managing inflammation. CRP, an inflammatory protein, can rise its normal level in response to infection or inflammation. As an acute phase protein, CRP increases by more than 25% during inflammatory conditions, including rheumatoid arthritis, etc. However, relying solely on these inflammatory markers does not provide sufficient predictive value for diagnosing acute appendicitis [8,9].

In addition to the previously mentioned inflammatory markers, there are also other cost-effective and easily measured markers of inflammation that are utilized in the diagnosis of acute appendicitis. The RDW (Red Cell Distribution Width) is an easily and automatically calculated indicator of the variability in red blood cell size. It is highly reproducible and can be easily obtained as part of routine blood exams without any extra cost. The RDW reflects the degree of variation (anisocytosis) in the size of circulating red blood cells. The last few years’ research has started to focus on this index for diagnosing acute appendicitis, and it may eventually be helpful in differentiating between uncomplicated and complicated cases of this condition [10]. Mean Platelet Volume (MPV) has been proposed as another potential biomarker for acute appendicitis in adults, although its role in pediatric cases remains unclear. However, recent meta-analyses have showed the way for its use on identifying complicated cases of the disease in the pediatric population [11].

Neutrophil-to-Lymphocyte ratio (NLR) and Platelet-to-Lymphocyte ratio (PLR) are recognized as cost-effective and easily quantifiable indicators of inflammation. Neutrophilia and lymphopenia are considered fundamental mediators of systemic inflammation. An elevated difference between Neutrophils and Lymphocytes indicates the intensity of the inflammatory response. As a result, the Neutrophil-to-Lymphocyte ratio (NLR) has been utilized as a marker in various pathological conditions. Both the NLR and PLR offer insights into immune and inflammatory pathways that have been investigated as potential predictors of the severity of appendicitis. In the past decade, both indicators have been thoroughly investigated, particularly in adult populations, for their potential in predicting the severity of acute appendicitis [12,13]. This study aims to assess their efficacy in differentiating between uncomplicated and complicated cases prior to surgical intervention. The analysis contributes to the existing literature, especially concerning pediatric patients. These markers serve as valuable tools for clinicians in guiding treatment decisions and establishing prognostic value, thereby facilitating the enhanced monitoring of patients and prioritizing those requiring surgical intervention. They also play a significant role in the diagnosis of pediatric patients who do not routinely undergo CT for establishing final diagnosis.

To sum up, acute appendicitis involves a broad spectrum of manifestations, ranging from simple inflammation of the appendix to perforation of the organ, which may be accompanied by localized or generalized peritonitis. In many cases, establishing a definitive diagnosis in younger patients poses a considerable challenge for clinicians. The surgeon is often called upon to determine whether to proceed with surgical intervention, even when the patient’s clinical presentation is not overtly alarming, in order to avoid complications associated with the condition. Thus, preoperative differentiation between uncomplicated and complicated disease is necessary for the physician to assess the urgency of surgical intervention, and, ultimately, to inform the guardians about the patient’s postoperative course, mortality rates, length of hospital stay, and duration of intravenous antibiotic treatment [14]. Complicated appendicitis in the pediatric population occurs in approximately 30% of cases, with prevalence rates ranging from 20% to 74%, with the highest rates being observed in younger patients. Advanced disease is associated with increased morbidity and a higher risk of postoperative complications [15].

## 2. Materials and Methods

### 2.1. Primary and Secondary Outcomes

The primary aim of the present statistical analysis is to present the basic epidemiological data (e.g., age and sex) concerning the various stages of acute appendicitis, while the inflammation proceeds, in the pediatric population. More specifically, through the study of two inflammatory markers, NLR (Neutrophil-to-Lymphocyte ratio) and PLR Platelet-to-Lymphocyte ratio), the ultimate outcome is to determine their sensitivity and specificity in distinguishing between patients with complicated and uncomplicated disease. In this way, we will examine whether they can be used in the monitoring of patients treated conservatively for uncomplicated disease. Apart from that, they will serve as a useful tool for the clinician in assessing the severity of the disease prior to surgery, potentially replacing other widely used inflammatory markers.

### 2.2. Design

This statistical analysis is based on data obtained from the records of our Pediatric Surgery University Clinic. A retrospective review was performed based on all appendectomies conducted on pediatric patients under the age of 16, with the youngest recorded patient being 17 months old. Specifically, the initial sample was extracted from the clinic’s records between January 2020 and December 2022, comprising a total of 341 patients who underwent both open and laparoscopic appendectomies. The Ethical Committee of our hospital approved our study on the 5 November 2019 (approval code: 329/5 November 2019).

### 2.3. Study Population, Sample, and Criteria Selection

The records of 341 patients were accessed from the clinic’s database, and the following data were initially documented: age, sex, and inflammation stage of the disease, as well as the values of Neutrophils, Platelets, and Lymphocytes. The stage of inflammation was related to the progression of the disease as assessed by the surgeon. Patients were classified into two categories based on age. The first group involved all patients under the age of 10 (Group H1), while the second group included those aged between 10 and 16 years old (Group H2). This age stratification was conducted for the better presentation of statistical data. Our Pediatric Surgery Clinic conducts surgical procedures on patients ranging from newborns to 16 years of age. The phase of inflammation was further subdivided into the following categories: (a) acute phase, (b) abscess, (c) gangrenous, (d) peritonitis, (e) plastron, and (f) negative appendectomies. This classification was based on the WSES guidelines established in 2020. According to the international literature, the disease phases are acute → abscess → gangrenous → localized or generalized peritonitis. The term appendiceal plastron refers to encapsulation of the organ by omental tissue. The “negative appendectomy” contains patients who met the clinical criteria for appendectomy, but no visible inflammation of the organ was identified during the procedure, prompting the investigation of alternative causes for the abdominal pain, such as the presence of mesenteric lymph nodes, a Meckel’s diverticulum, or pathology of the internal genital organs in female patients. Furthermore, the absolute values of Neutrophils, Lymphocytes, and Platelets were documented from the patients’ records. Only patients for whom laboratory tests were conducted within 24 h prior to surgery were included in our study.

To sum up, the initial sample comprised of 341 patients aged up to 16 years old who underwent appendectomy, during the three-year period from 2020 to 2022. However, due to a lack of data regarding demographic characteristics and measurements of Neutrophil, Lymphocyte, and Platelet values, the sample was subsequently reduced to 288 patients. Of these, 14 had incomplete data concerning age and sex, while 39 lacked the necessary laboratory values. Additionally, patients diagnosed with appendiceal plastron were excluded from the study (6 out of 341, representing 1.7% of the total). Furthermore, no cases regarding other inflammatory conditions such as inflammation of Meckel’s diverticulum or inflammatory bowel diseases were included. Consequently, the statistical analysis was conducted with a final sample of 282 patients. Furthermore, patients with neoplasia of the appendix were not included in this study.

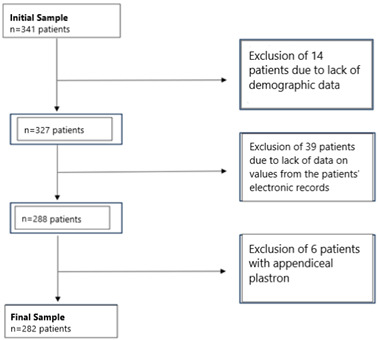



### 2.4. Statistical Analysis

A principal tool utilized for the execution of the present statistical analysis was the SPSS application, Statistical Package for the Social Sciences, for Windows, specifically version 25.0. The markers assessed in this analysis, namely the Neutrophil-to-Lymphocyte ratio (NLR) and the Platelet-to-Lymphocyte ratio (PLR), were evaluated using the Receiver Operating Characteristic (ROC) curve. The area under this curve (AUC, area under the curve) signifies the accuracy in differentiating between uncomplicated and complicated disease. A *p*-value of less than 0.05 was deemed statistically significant.

## 3. Results

### 3.1. Demographic Characteristics: Age and Gender

The data of 288 patients were analyzed as follows. The mean age of these patients was 10 years and 9 months, with a standard deviation of 3 years and 3 months (10.9 ± 3.3). The sample was categorized into two age groups, as previously indicated, with approximately equal numbers of patients. The first group (Group H1) involved patients up to 10 years of age, which totaled 130 patients (45.1%), while the second group (Group H2) included 158 patients (54.9%). The male patients of our study totaled 179 (62.2%), while the female patients totaled 109 (37.8%).

### 3.2. Progression of Inflammation/Disease

Our patients were categorized by the observed disease phase during surgery based on the surgeon’s evaluation and histological findings. Interestingly, 35.7% of patients were associated with the “Abscess” phase, while 6.94% underwent surgery for peritonitis. The acute phase accounted for 30.2%, and the gangrenous phase for 18.0%. According to the WSES Surgical Society classification, these stages are categorized into uncomplicated and complicated appendicitis based on algorithms published in 2020. Thus, within a sample size of 262 patients, uncomplicated disease related to 72.5%, while 27.5% of cases were classified as complicated.

### 3.3. Inflammatory Markers

Table 1 delineates the descriptive characteristics of the values for Neutrophils, Lymphocytes, and Platelets within the sample of 288 participants, as recorded in their medical records. The normal values, according to our laboratory data, are defined as follows:

1. Neutrophils: normal values (N.V.) → 1.1 − 6.6 (×10^3^/μL)
2. Lymphocytes: N.V. → 1.0 − 9.0 (×10^3^/μL)
3. Platelets: N.V. → 140 − 440 (×10^3^/μL).

The two primary markers of the study were established, which have been extensively examined in recent years within the international literature regarding adults and are now being investigated in pediatric patients. The first variable is the Neutrophil-to-Lymphocyte ratio (NLR), as indicated in the subsequent charts, and the second is the Platelet-to-Lymphocyte ratio (PLR). Table 2 presents the descriptive statistics for these two variables based on the phase of inflammation. An increase in the values of both ratios is observed with the progression of the inflammatory phase of the appendix. On average, the NLR was 11.50 ± 8.87 in patients with peritonitis, whereas the corresponding value was significantly lower in children with acute wall inflammation. Concerning the second parameter, the PLR, a similar increase in value is noted while the disease progresses. In patients without detected wall inflammation, the average PLR was 161.31 ± 116.35.

**Figure 1:**
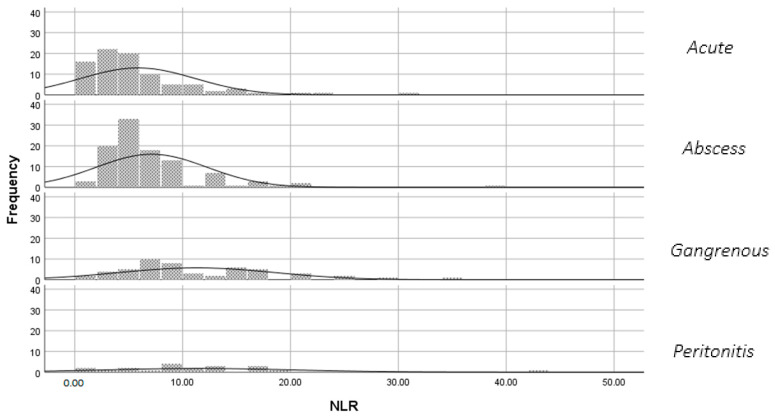
Variation of the NLR according to the phase of inflammation.

**Figure 2:**
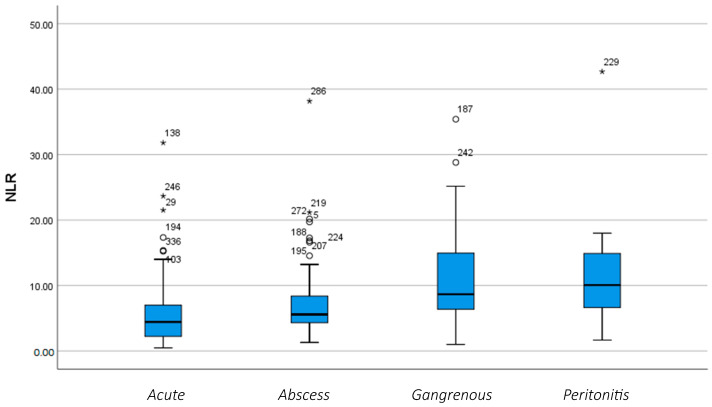
Variation of the NLR according to the phase of inflammation. (*, o: These symbols refer to patients who deviate from standard values).

The afore-mentioned statistical Figure 1 and Figure 2 provide a detailed illustration of the Neutrophil-to-Lymphocyte ratio (NLR) as inflammation progresses from the acute phase to more advanced stages.

**Figure 3:**
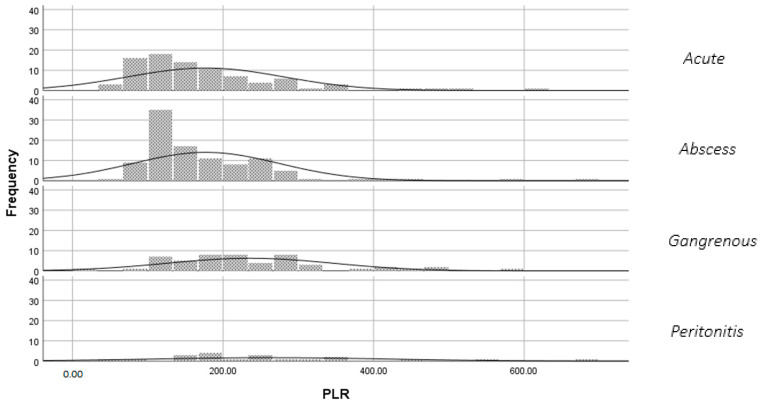
Variation of the PLR according to the phase of inflammation.

**Figure 4:**
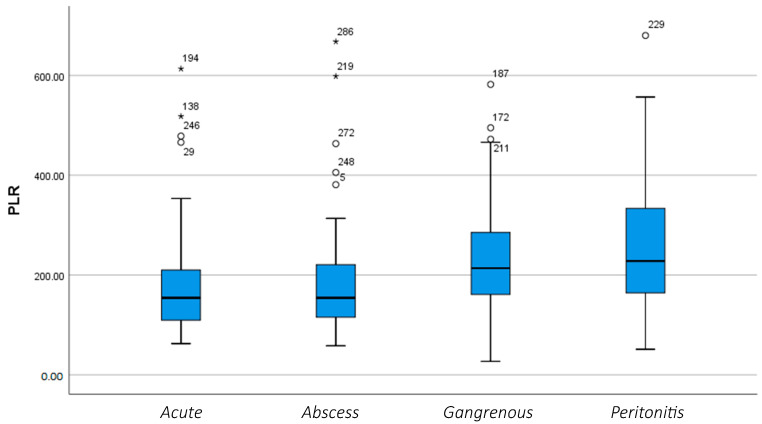
Variation of the PLR according to the phase of inflammation. (*, o: These symbols refer to patients who deviate from standard values).

According to the data, Figure 3 and Figure 4 demonstrate the average Platelet-to-Lymphocyte ratio (PLR) that also exhibits an increase in the advanced phases of inflammation. The standard deviations similarly rise, indicating greater variability in PLR values among patients.

Table 3 outlines the distinction between uncomplicated and complicated appendicitis. In the advanced stages of disease, the two ratios are elevated in comparison to the initial stages of inflammation.

**Figure 5:**
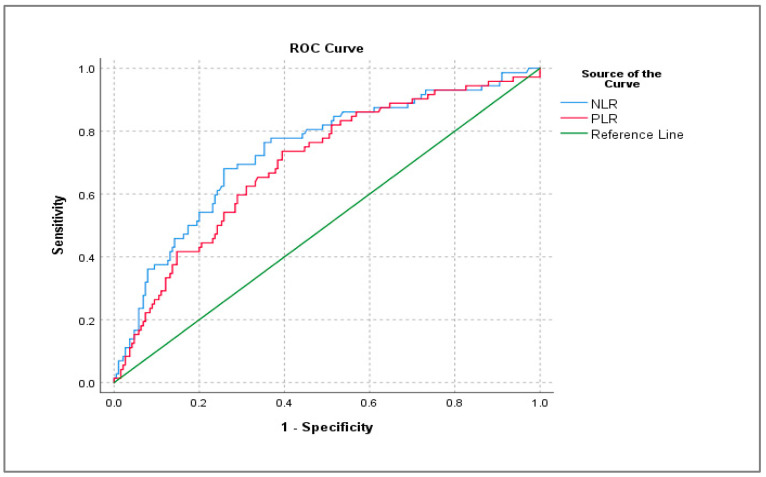
ROC curve for the entire sample of the study.

An ROC (Receiver Operating Characteristic) analysis was conducted utilizing the SPSS 29.0.2.0 statistical software. The following graphical representation in Figure 5 illustrates sensitivity as a function of the inverse of specificity, namely, 1-Specificity. The area under the ROC curve (AUC) shows the reliability of the marker between uncomplicated and complicated appendicitis.

The results presented in Table 4 indicate that the area under the ROC curve (AUC) is 0.736, which corresponds to 73.6% of the area, demonstrating that the model exhibits adequate quality in differentiating between patients with uncomplicated and complicated appendicitis. It is worth mentioning that a higher AUC value would signify improved overall performance of the model.

**Figure 6:**
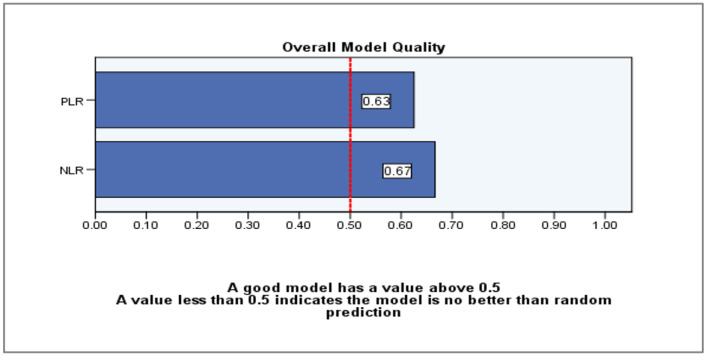
ROC model quality for the entire sample.

As depicted in Figure 6, the quality of our data is deemed reliable, as each parameter possesses a value more than 0.5.

**Figure 7:**
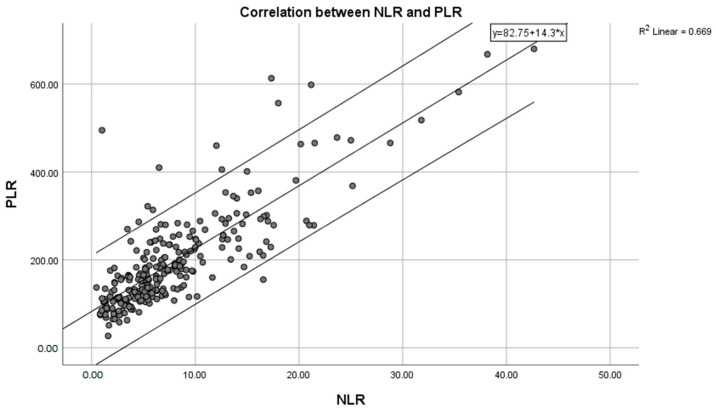
Correlation of two parameters of the study in a linear curve.

In the present ROC analysis, the cut-off point for the Neutrophil-to-Lymphocyte ratio (NLR) was established at 7.92, depicting a sensitivity of 62.5% and a specificity of 74.2%. For instance, when patient X has an NLR value exceeding 7.92, the probability of having complicated appendicitis is 62.5% among all cases of complicated appendectomies. Similarly, the cut-off point for the Platelet-to-Lymphocyte ratio (PLR) was determined to be 180.57, with a sensitivity of 61.1% and a specificity of 68.9%. For example, when patient Y presents a PLR value less than 180.57, the probability of having uncomplicated appendicitis is 68.9% among all cases of uncomplicated appendectomies. However, it is advisable to utilize both ratios in conjunction for disease prediction. The correlation between the two inflammatory markers in our study is illustrated in the following graph (Figure 7). The curve estimation indicated that the Neutrophil-to-Lymphocyte ratio (NLR) and the Platelet-to-Lymphocyte ratio (PLR) exhibit a notably strong linear relationship, as showed by the equation PLR = 82.75 + 14.3 × NLR (R^2^ = 0.669, *p* < 0.001).

**Figure 8:**
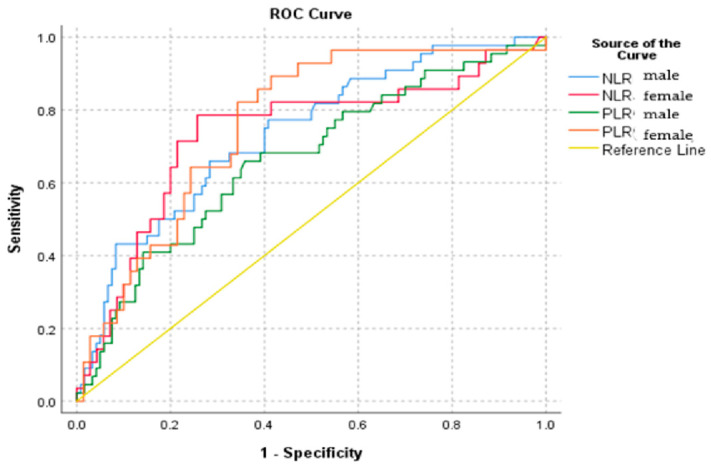
ROC curve by gender.

**Figure 9:**
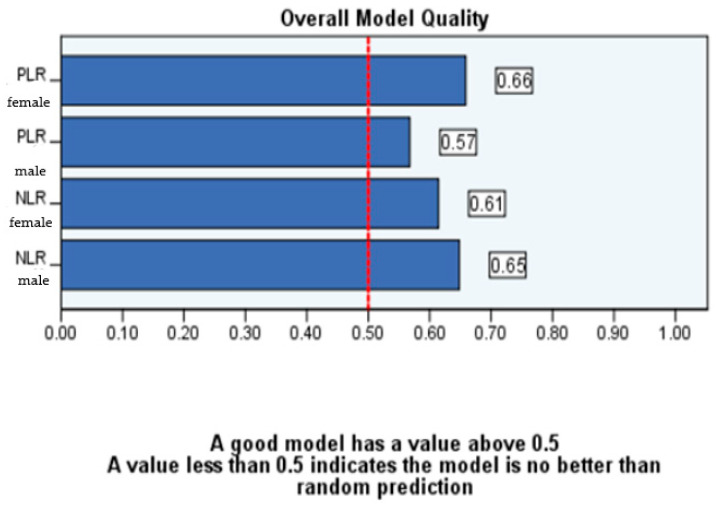
ROC model quality by gender.

The ROC analysis was similarly separately conducted as far as gender and age are concerned. It is noteworthy that, in female patients, the PLR demonstrates greater reliability compared to male patients, as shown by a higher value of the area under the curve (Table 5, Figure 8 and Figure 9).

**Figure 10:**
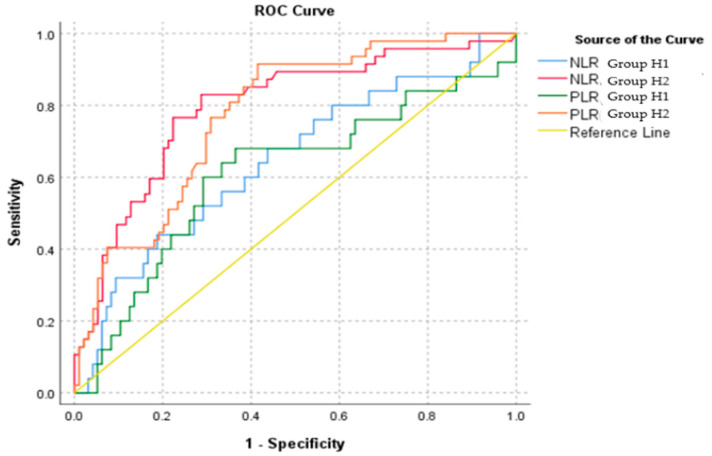
ROC curve by age group.

**Figure 11:**
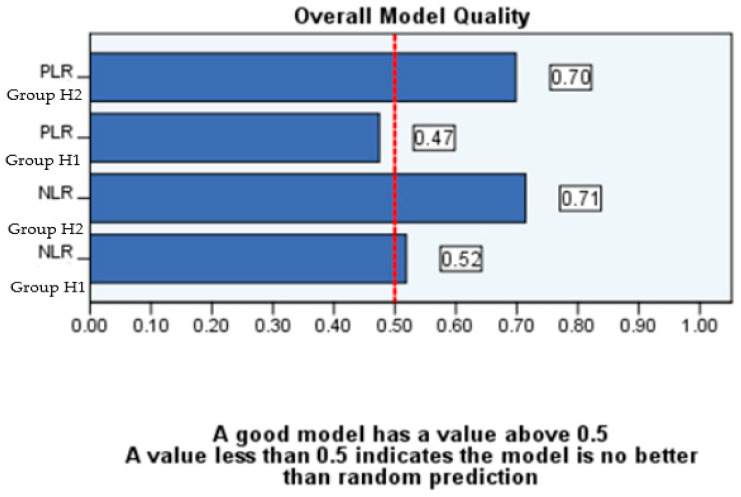
ROC model quality by age group.

However, as shown by the results presented in Table 6 and Figure 10 and Figure 11, in which ROC analysis was performed by age group, no definitive statistical conclusions can be withheld regarding patients under the age of 10. Consequently, this particular model predicts cases of complicated disease with greater reliability among patients aged between 10 and 16 years old (Group H2).

Finally, it is worth mentioning that all continuous variables regarding this statistical analysis were tested for the normality of data distribution using the Kolmogorov–Smirnov test. Continuous variables include Neutrophils, Lymphocytes, and Platelets, as well as the basic inflammation markers: NLR and PLR. Of these variables, only Neutrophils separately follow a normal distribution. The main markers (NLR and PLR) do not follow a normal distribution, so analyses for non-parametric tests, such as the Mann–Whitney test, were used.

## 4. Discussion

Our findings demonstrate a correlation between the Neutrophil-to-Lymphocyte ratio (NLR) and the Platelet-to-Lymphocyte ratio (PLR) in differentiating between uncomplicated and complicated appendicitis in children. Specifically, the NLR showed a sensitivity of 62.5% and a specificity of 74.2%. In comparison, the PLR had a sensitivity of 61.1% and a specificity of 68.9%. These results were comparable to the more widely used C-reactive protein (CRP), which exhibited a sensitivity of 70% and a specificity of 71.8% [16]. McGowan et al. reported that CRP exhibited a sensitivity ranging from 46% to 94% and a specificity between 32% and 84%, depending on the cut-off values [17,18].

There has been a discernible trend towards the conservative management of uncomplicated conditions, wherein the NLR and PLR assume a significant role regarding the monitoring of treatment in patients with uncomplicated appendicitis [19]. Research indicates that, as the inflammation of the appendix advances, the number of Lymphocytes diminishes, while the number of Neutrophils increases. This is explained by the elevation of the NLR as the disease approaches the gangrenous stage [20].

In 1995, Goodman et al. [21] first introduced the Neutrophil-to-Lymphocyte ratio as a diagnostic tool in the international literature. They demonstrated that values exceeding 3.5 were associated with an increased possibility of diagnosing acute appendicitis [21]. Subsequent studies have emphasized that this ratio can be employed as an indicator of inflammation and plays a crucial role in differentiating between uncomplicated and complicated appendicitis [22]. According to Markar et al. [23], the NLR appears to demonstrate greater diagnostic sensitivity than both white blood cell counts and C-reactive protein (CRP) in the diagnosis of this condition. Furthermore, the statistical analysis conducted by Shimizu et al. indicates that, as inflammation progresses, the NLR exhibits a notable increasing trend, similar to the data within this particular study [23,24].

One of the most recent statistical analyses in the literature concerning the pediatric population related to a three-year study involving 232 patients. In this study, 54.19% of the patients were male, while 45.81% were female, contrary to our analysis, which indicated that 62.2% were boys and 37.8% were girls. Notably, 39.09% of boys and 21.51% of girls were diagnosed with complicated disease, compared to our findings, where the rates were 24.5% and 25.6%, correspondingly [25]. The mean values of the NLR reported by La Cruz-Vallejo et al. [26] were measured at 10.73 ± 5.28, whereas, in this present study, they were 10.76 ± 4.79. Similarly, the values for the PLR were recorded as 1.49 ± 0.83 and 1.89 ± 0.93, respectively. The area under the curve (AUC) was calculated to be 0.74 for the two ratios, contrary to our study, in which the AUC for the NLR was 0.736, while for the PLR, it was 0.694. Furthermore, the NLR for values exceeding 10.4 exhibited a sensitivity of 77.78% and a specificity of 67.14% in distinguishing between uncomplicated and complicated disease; in our study, the sensitivity was 62.5% and the specificity was 74.2% for values greater than 7.92. Additionally, for the second inflammatory index, the PLR, La Cruz-Vallejo et al. reported a sensitivity of 77.78% and a specificity of 63.57% for values above 284. In our analysis, the most representative value for this inflammatory index was 180.57, with a sensitivity of 61.1% and a specificity of 68.9% [26].

In a seven-year study involving 701 patients, Ayeni et al. [16] demonstrated the sensitivity and specificity of two inflammatory ratios in distinguishing between complicated and uncomplicated disease. They reported that values exceeding 193.67 for the PLR, yielded a sensitivity of 64% and a specificity of 61%. The variability of the ratios described in the literature is large due to population heterogeneity regarding geographic and national levels [16]. When combining the two markers simultaneously, this can indicate severe inflammatory cases and serve as a valuable tool in clinical decision making. In our study, the two ratios were correlated, following the equation PLR = 82.75 + 14.3 × NLR.

One of the most recent meta-analyses, which aims to review the international literature on the Neutrophil-to-Lymphocyte ratio, was published in 2019 and involves data from 11 studies with 8.914 adult patients. This analysis underlines that the cut-off point for the NLR in differentiating between uncomplicated and complicated disease is 8.8, yielding a sensitivity of 76.92% and a specificity of 100%, as reflected in an area under the curve (AUC) of 0.91. Additionally, the study calculates that the cut-off point for distinguishing between cases of non-inflammation of the appendix and uncomplicated disease is 4.7, with a sensitivity of 88.89% and a specificity of 90.91% [27]. The cut-off value for the NLR reported by Kahramanca et al. was significantly lower, at 5.74, yielding a sensitivity of 70.8% and a specificity of 48.5%. Contrary to our study, the cut-off value approached 7.92, with sensitivity of 62.5% and a specificity of 74.2% [28].

The findings of this study demonstrate that the NLR and PLR are significantly elevated in cases of advanced appendiceal inflammation compared to uncomplicated phases of the disease. More specifically, the sensitivity and specificity of the NLR for diagnosing the disease range from 63% to 90% and from 57% to 89%, respectively [26,27]. In our study, 27.5% of the patients presented with a complicated form of the disease, a substantially lower percentage than the 81% reported by Esquivel-Esquivela et al. In other statistical studies, the incidence of complicated disease ranges from 11% to 68% [28,29]. The diagnostic accuracy of the NLR and PLR in differentiating between uncomplicated and complicated disease exhibits considerable variability in the literature. The ability of the NLR to distinguish between simple and complicated appendicitis reflects the area under the curve when ranging from 0.66 to 0.84, with cut-off points between 4.8 and 10.4 and sensitivities from 67% to 85% [26,28,29]. In the present statistical analysis, the area under the curve for the NLR was determined to be 0.736, with a cut-off point of 7.92 and a sensitivity of 62.5%. The ease of measuring these two markers, in concomitant with their low cost in comparison to already-established inflammatory markers such as C-reactive protein (CRP), underlines the increasing interest for their use the recent years. This analysis is considered to be one of few studies in the international literature that thoroughly examines these markers according to appendiceal perforation, and, more specifically, in advanced forms of the disease within the pediatric population. The aforementioned markers are easily measured in the Emergency Department and are relatively cost-effective, as they can be assessed through the patient’s complete blood count.

This study is retrospective and single-center, and its results should be compared with prospective future statistical analyses. Indeed, it presents data for distinguishing between uncomplicated and complicated appendicitis. A better interpretation of the results could be achieved by studying these indicators in differentiating between the absence of disease and simple inflammation of the appendix. Cases of the localized inflammation of the appendix due to omentum or another segment of the intestine (plastron) were excluded. There is a clear need for a larger multicentric study, as this investigation was conducted in a single hospital institution, relying on data and measurements from one microbiological laboratory. In future, research should be published with a larger number of patients.

## 5. Conclusions

The markers of Neutrophil-to-Lymphocyte (N/L) and Platelet-to-Lymphocyte (P/L) ratios are proven to be particularly valuable for the monitoring of patients receiving conservative treatment, especially during the early stages of inflammation. Ultimately, these ratios may serve as diagnostic tools for identifying patients with acute appendicitis in situations where clinical judgment is uncertain, particularly when imaging modalities such as ultrasound and computed tomography are not available. The findings from our study indicate that these markers demonstrate relatively good sensitivity and specificity, enabling their use in differentiating patients with clinically confirmed acute appendicitis in healthcare institutions with a limited number of operating rooms. This study represents a significant contribution to the foundation of laboratory diagnosis for acute appendicitis, suggesting that, in conjunction with other analyses, these inflammatory parameters may ultimately replace other markers such as C-reactive protein (CRP).

In summary, the NLR exceeding 7.92 facilitates the differentiation between uncomplicated and complicated forms of acute appendicitis, showing a sensitivity of 62.5% and a specificity of 74.2%. Likewise, PLR values greater than 180.57 support the diagnosis, with a sensitivity of 61.1% and a specificity of 68.9%.

## Figures and Tables

**Table 1:** Descriptive summary characteristics of Neutrophils, Lymphocytes, and Platelets of the participants.

	Neutrophils	Lymphocytes	Platelets
Mean Value	10.76	-	-
Standard Deviation	4.79	0.93	82.08
Median Value	-	1.78	282.0
Dominant value	8.1	1.6	277
Interval	31.8	6	602
Minimum Value	0.60	0.30	23
Maximum Value	32.40	6.30	625

**Table 2:** Consolidated descriptive characteristics of the study according to the phase of inflammation.

Inflammation Stage	Sex		Age Mean	NLR (Neutrophil-to-Lymphocyte Ratio)		PLR (Platelet-to-Lymphocyte Ratio)	
	Male	Female		Median Value	Standard Deviation	Median Value	Standard Deviation
Plastron	5	1	7.0 (5.1)	5.15	4.17	164.91	69.87
Negative	10	10	11.4 (2.5)	4.93	4.80	161.31	116.35
Acute	47	40	10.5 (3.2)	5.85	5.33	176.73	104.07
Abscess	73	30	11.2 (2.8)	7.13	5.15	177.27	97.70
Gangrenous	36	16	10.78 (3.7)	11.32	7.20	235.96	110.69
Peritonitis	8	12	9.6 (4.0)	11.50	8.87	262.67	156.94

**Table 3:** Descriptive characteristics of the study ratios according to the phase of inflammation.

Inflammation Stage	Sex		Age Mean	NLR (Neutrophil-to-Lymphocyte Ratio)		PLR (Platelet-to-Lymphocyte Ratio)	
	Male	Female		Median Value	Standard Deviation	Median Value	Standard Deviation
Uncomplicated	120	70	10.9 (3.0)	6.54	5.26	177.02	100.40
Complicated	44	28	10.4 (3.8)	11.37	7.64	243.38	124.65

**Table 4:** Results of the ROC analysis for the entire sample.

Markers	AUC-Area Under Curve	Standard Error	Asymptotic Sig.	Confidence Level 95%	
				Upper Limit	Lower Limit
NLR (Neutrophil-to-Lymphocyte ratio)	0.736	0.035	0.000	0.666	0.805
PLR (Platelet-to-Lymphocyte ratio)	0.696	0.036	0.000	0.625	0.767

**Table 5:** Results of the ROC analysis by gender.

Markers		AUC-Area Under Curve	Standard Error	Asymptotic	Confidence Level 95%	
					Upper Limit	Lower Limit
NLR (Neutrophil-to-Lymphocyte ratio)	Male	0.734	0.044	0.000	0.648	0.819
	Female	0.734	0.061	0.000	0.614	0.853
PLR (Platelet-to-Lymphocyte ratio)	Male	0.663	0.049	0.001	0.568	0.758
	Female	0.760	0.052	0.000	0.659	0.861

**Table 6:** Results of the ROC analysis by age group.

Markers		AUC-Area Under Curve	Standard Error	Asympomatic	Confidence Level 95%	
					Upper Limit	Lower Limit
NLR (Neutrophil-to-Lymphocyte ratio)	≤10 years	0.644	0.064	0.024	0.519	0.769
	>10 years	0.795	0.041	0.000	0.714	0.876
PLR (Platelet-to-Lymphocyte ratio)	≤10 years	0.609	0.069	0.113	0.474	0.744
	>10 years	0.776	0.040	0.000	0.698	0.854

## Data Availability

The original contributions presented in the study are included in the article, further inquiries can be directed to the corresponding author.
